# Protective Effect of Thyme and Chestnut Honeys Enriched with Bee Products against Benzo(a)pyrene-Induced DNA Damage

**DOI:** 10.3390/ijerph192416969

**Published:** 2022-12-17

**Authors:** Vanesa Sánchez-Martín, Ana I. Haza, Amaia Iriondo-DeHond, María Dolores del Castillo, Xavier F. Hospital, Manuela Fernández, Eva Hierro, Paloma Morales

**Affiliations:** 1 Departamento de Nutrición y Ciencia de los Alimentos, Sección Departamental de Nutrición y Ciencia de los Alimentos, Facultad de Veterinaria, Universidad Complutense, 28040 Madrid, Spain; 2 Food Bioscience Group, Department of Bioactivity and Food Analysis, Instituto de Investigación en Ciencias de la Alimentación (CIAL) (CSIC-UAM), 28049 Madrid, Spain; 3 Departamento de Farmacia Galénica y Tecnología de los Alimentos, Sección Departamental de Farmacia Galénica y Tecnología de los Alimentos, Facultad de Veterinaria, Universidad Complutense, 28040 Madrid, Spain

**Keywords:** antioxidant, benzo(a)pyrene, chemopreventive agent, comet assay, DNA damage, genotoxicity, honey, royal jelly, propolis

## Abstract

The aim of the present study was to validate the cytotoxicity, genotoxicity, and preventive potential against benzo(a)pyrene (BaP)-induced DNA damage of nine samples of thyme and chestnut honeys enriched with bee products (royal jelly and propolis, 2–10%). Cell viability was determined by the MTT [3-(4,5-dimethylthiazol-2-yl)-2,5-diphenyltetrazolium bromide] assay (0–250 mg/mL) to select nontoxic concentrations, and DNA damage (0.1–10 μg/mL) was evaluated by the alkaline single-cell gel electrophoresis or comet assay. Treatment with honey samples or royal jelly and propolis did not affect the viability of HepG2 cells up to 100 and 50 mg/mL, respectively. Treatment with 100 μM BaP significantly increased (*p* ≤ 0.001) the levels of the DNA strand breaks. None of the tested concentrations (0.1–10 μg/mL) of the honey samples (thyme and chestnut), royal jelly, and propolis caused DNA damage *per se*. All tested samples at all the concentrations used decreased the genotoxic effect of BaP. In addition, all mixtures of thyme or chestnut honeys with royal jelly or propolis showed a greater protective effect against BaP than the samples alone, being the thyme and chestnut honey samples enriched with 10% royal jelly and 10% propolis the most effective (70.4% and 69.4%, respectively). The observed protective effect may be associated with the phenolic content and antioxidant capacity of the studied samples. In conclusion, the thyme and chestnut honey samples enriched with bee products present potential as natural chemoprotective agents against the chemical carcinogen BaP.

## 1. Introduction

The European Union (EU) is the second honey producer worldwide after China, and Spain is the country with the largest honey production [1]. In addition to honey, Spain produces a variety of apiculture products, such as pollen, propolis, royal jelly, and beeswax [2]. Although the EU is an important honey producer (25,500 tons exported in 2021), EU member states imported 173,400 tons of honey in 2021, which came mostly from Ukraine (31%) [3]. In the context of the Ukraine war and climate change, sourcing significant volumes of honey required to replace the Ukrainian honey market is a great challenge [4]. Therefore, more efforts are needed to support local honey production. On the other hand, during the COVID-19 pandemic, the global honey market exhibited an increasing demand, and consumers are now asking for immunity-boosting and healthy food products [5,6]. In addition to the known effects of honey on the immune system, it has also shown antimicrobial, antioxidant, anticancer, antihyperlipidemic, and cardioprotective properties [7]. Consequently, the interest in exploring the health-promoting properties of not only honey, but also other bee products, such as royal jelly and propolis, has recently grown [8].

Honey contains more than 180 types of different compounds, including water, sugars, free amino acids, proteins, essential minerals, vitamins, and various phytochemicals, such as phenolic acids, flavonols, and alkaloids [9]. These phytochemicals are mainly responsible for the antioxidant capacity of honey and its ability to scavenge free radicals [10]. Propolis or “bee glue”, composed of resin, wax, balsam, and other phytochemicals [11], has also shown a capacity to scavenge hydroxyl and superoxide radicals that cause cell damage and related diseases. These properties are associated with the presence of phenolic compounds, such as *p*-coumaric (1.2–12.2 mg/g) and ferulic acid (0.3–11.0 mg/g), and flavonoids (pinocembrin, catechin, caffeic acid phenethyl ester, and galangin) present in propolis [12]. Lastly, royal jelly or “bee milk” is an acidic colloid composed of water (60–70%), proteins (9–18%), sugars (7–18%), lipids (3–6%), and other compounds (0.8–3%), such as free amino acids and fatty acids [13,14]. Due to its antimicrobial, antioxidant, antiaging, and immunomodulatory properties, it is the most studied bee product [14].

In the past years, the exposure to polycyclic aromatic hydrocarbons (PAHs), more specifically to benzo(a)pyrene (BaP), is more common than ever before [15]. BaP is a crystalline, aromatic hydrocarbon that consists of five fused benzene rings and is formed during the incomplete combustion or pyrolysis of organic matter [16]. It is primarily found in gasoline and diesel engine exhaust, petroleum asphalt, charcoal-broiled foods, amino acids, fatty acids, and carbohydrate pyrolysis products, among others [17]. Humans are exposed to BaP mainly by cigarette smoke and foods contaminated from environmental sources, industrial food processing (heating, drying, and smoking processes), and from certain home cooking practices (grilling and roasting) [18]. According to the International Agency for Research on Cancer (IARC), BaP is a Group I carcinogen to humans [19]. BaP is also genotoxic, mutagenic, epigenotoxic, teratogenic, and neurotoxic, and it impairs fertility [20,21]. One of BaP’s mechanisms of action is its pro-oxidative effects that lead to lipid peroxidation, protein carbonylation, and DNA damage [15].

Recent studies have shown the protective effect of honey and other bee products on induced DNA damage [22,23,24]. Stingless bee honey has been used by traditional medicine practitioners for many years and has recently shown protective effects against hydrogen peroxide (H_2_O_2_)-induced cell death and DNA damage in human lymphoblastoid cells [23]. Propolis is also able to protect induced oxidative DNA damage in human liver cells through reactive oxygen species (ROS) scavenging [22]. In addition, royal jelly has presented antigenotoxic and antimutagenic properties *in vivo* in mice treated with methyl methanesulfonate [24]. However, there is limited information regarding the effect of monofloral honey samples and their combination with propolis and royal jelly on DNA damage. Therefore, the aim of the present study was to validate the cytotoxicity, genotoxicity, and preventive potential against BaP-induced DNA damage of nine samples of thyme and chestnut honeys enriched with bee products (royal jelly and propolis, 2–10%).

## 2. Materials and Methods

### 2.1. Chemicals

Benzo(a)pyrene (BaP), dimethyl sulfoxide (DMSO), GelRed^®^1X, and low-melting-point agarose (LMP) were purchased from Sigma-Aldrich (St. Louis, MO, USA). BaP was dissolved in sterile DMSO. The stock solutions were stored deep-frozen (−80 °C). All other chemicals and solvents were of the highest grade commercially available. Dulbecco’s Modified Eagle Medium (DMEM), fetal calf serum, penicillin and streptomycin, and L-glutamine were purchased from Gibco Laboratories (Life Technologies, Inc., Gaithersburg, MD 20884-9980, USA).

### 2.2. Raw Materials

Thyme and chestnut honeys were obtained directly from beekeepers of each region. The type and geographic region of honey samples, as well as the family, scientific, and common names of the plants that form the basic flora of the honey samples, royal jelly and propolis, are shown in Table 1. Samples were obtained in January 2021. In our previous study, the analysis of the physicochemical parameters of both honey samples showed values within the limits established by the Spanish Royal Decree Law (RD) 1049/2003 [25]. A honey sample is classified as monofloral if it contains pollen in quantities exceeding 45% of the remaining pollen identified. In any other case, a honey sample is characterized as heterofloral [26,27]. In our previous research work, the botanical origin of thyme and chestnut honeys was evaluated, and the percentage of pollen in thyme honey was 47.19% and 64.98% for chestnut honey [25]. Therefore, in this research work, two monofloral honeys were used, thyme honey and chestnut honey.

Thyme and chestnut honeys were mixed with royal jelly and propolis (2–10%). Samples (250 g) were homogenized in a laboratory blender, LB 400 W (with glass window in the door, blade speed (min^−1^): 240, fixed speed: 8 strokes/s). In order to optimize the extraction of antioxidant components and mixtures, propolis tincture was used and dissolved in 70% ecologic ethanol (La Alcoholera, La Rioja, Spain). To prepare honey, royal jelly, propolis, and mixture samples with 2 and 10% of royal jelly or propolis were weighed and diluted in sterile distilled water (1 g/mL). Then, sample solutions were sterilized by Millipore filtration (0.45 µm) and stored at −20 °C [25].

The 9 samples evaluated in the present work (Table 2) were previously selected from among 16 samples of honey mixtures with royal jelly and propolis (2–10%) for their high phenolic content and antioxidant capacity [25].

### 2.3. HepG2 Cells

HepG2 cells were provided by Unidad de Terapias Farmacológicas and Unidad de Genética Molecular (Instituto de Investigación de Enfermedades Raras, Instituto de Salud Carlos III, Madrid, Spain). Only cells of passages 10–17 were used in these experiments. Cells were cultured as a monolayer in DMEM supplemented with 10% *v*/*v* heat-inactivated fetal calf serum, 50 U/mL penicillin, 50 mg/mL streptomycin, and 1% *v*/*v* L-glutamine. Cell cultures were incubated at 37 °C and 100% humidity in a 5% CO_2_ atmosphere.

### 2.4. Cytotoxicity

Cell viability was determined by the MTT [3-(4,5-dimethylthiazol-2-yl)-2,5-diphenyltetrazolium bromide] assay (Cell Proliferation Kit I, Roche, Indianapolis, IN, USA) to select nontoxic concentrations of samples. First, HepG2 cells were cultured at a density of 1 × 10^5^ cells per well of a 96-well plate for 24 h. Then, cells were treated with honey and bee product samples (Table 2) at concentrations of 0–250 mg/mL for 24 h. Subsequently, cells were incubated in MTT Labeling Reagent for 4 h at 37 °C, and then 100 µL of solubilization solution were added. After 24 h, the optical density of each well was read at 620 nm (test wavelength) and 690 nm (reference wavelength) using a microplate reader. Experiments were carried out in triplicate (n = 16). Results were expressed as the percentage of viability (% cell survival) with respect to the control (medium-treated cells).

### 2.5. Analysis of DNA Damage (DNA Strand Breaks Induced by Honey Samples, Royal Jelly, and Propolis in the Alkaline Comet Assay)

The alkaline comet assay is a technically simple, sensitive assay to detect DNA damage, such as strand breaks [28]. This section includes the Minimum Information for Reporting Comet Assay (MIRCA), a standardized reporting checklist for the description of comet assay procedures and results as a tool to ensure that the comet assay is performed rigorously and reported comprehensively [28]. The comet assay was carried out according to the protocol of Olive et al. (1992) [29]. Briefly, HepG2 cells were plated onto a 24-well plate at a density of 1.5 × 10^5^ cells/mL in culture medium. Twenty-four hours after seeding, cells were exposed to thyme (TH) and chestnut (CH) honey samples and royal jelly (RJ) and propolis (PR) (0.1–10 µg/mL) for another 24 h at 37 °C and 5% CO_2_. Treatment with BaP (100 µM) was used as a positive control, and untreated cells (C_0_) were used as a negative control. After incubation, 12 µL of a suspension of 1.5 × 10^5^ cells was mixed with 70 µL of LMP agarose type VII (0.75% concentration in PBS) reaching a final concentration of agarose of 0.64%, distributed on slides (Diagnostic microscope slides, 3-well 14 mm, ER-203B-CE24, Thermo Scientific, Waltham, MA, USA) that had been precoated with LMP agarose type VII (0.30% concentration in PBS) and left to set on an ice tray. After solidification, cells were lysed in darkness for 1 h in a high salt alkaline buffer (2.5 M NaCl, 0.1 M EDTA, 0.01 M Tris, 1% Triton X-100, pH 10). After incubation, slides were placed in electrophoresis buffer (0.3 M NaOH, 1 mM EDTA, pH 13, cooled in a refrigerator) in darkness for 40 min. Electrophoresis was performed in a cold storage room, in darkness, in a Bio-Rad subcell GT unit containing the same buffer, for 30 min at 25 V. After electrophoresis, the slides were neutralized using 0.4 M Tris pH 7.5 and fixed in methanol. Subsequently, DNA was stained with GelRed^®^1X in Tris acetate EDTA (TAE 1×) for 5 min and examined in a fluorescence microscope (microscope magnification 10×) (OLYMPUS BH-2) connected to a computerized image analysis system (Komet 5.1). Results were expressed as percentage of Tail DNA.

### 2.6. Analysis of DNA Damage Induced by a Simultaneous Treatment with BaP and Honey Samples, Royal Jelly, Propolis, and Their Mixtures in the Alkaline Comet Assay

HepG2 cells were plated onto 24-well plates at a density of 1.5 × 10^5^ cells/mL culture medium. Twenty-four hours after seeding, the samples (Table 2) at 0.1–10 µg/mL were added to the wells, and plates were incubated for 24 h at 37 °C and 5% CO_2_. After incubation, cells were simultaneously treated with BaP (100 µM) and different concentrations of samples for another 24 h at 37 °C and 5% CO_2_. After the combined treatment of cells with BaP and samples, cells were processed as described above.

### 2.7. Statistical Analysis

Images of 50 randomly selected cells per concentration were evaluated, and the test was carried out three times. The reported Tail DNA is the mean ± standard error (SE) of three independent experiments. One-way analysis of variance (ANOVA) was carried out, and statistical comparisons of the different treatments were performed using Tukey’s test. Values of *p* < 0.05 were considered statistically significant. All statistical analyses were performed using the Statgraphics Centurion 19 software (Statgraphics Technologies, Inc. The Plains, VA, USA).

## 3. Results and Discussion

### 3.1. Evaluation of Cytotoxicity of Samples

**Figure 1:**
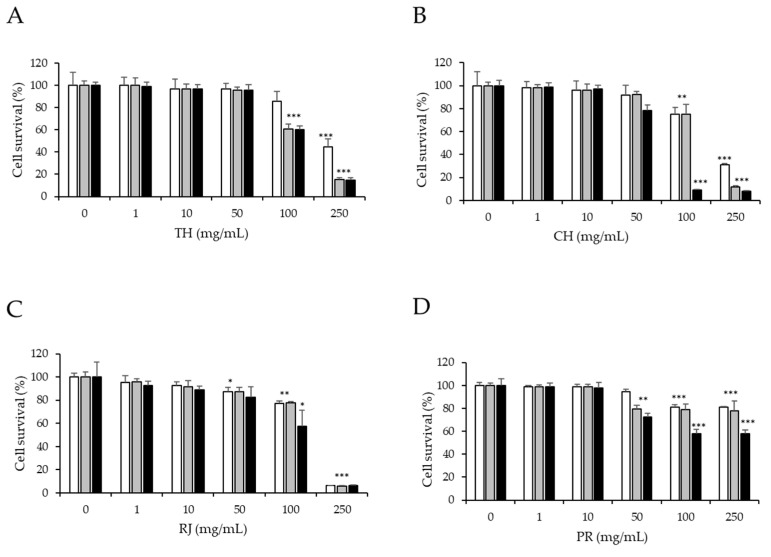
Effect of (**A**), thyme honey (TH, sample 1); (**B**), chestnut honey (CH, sample 6); (**C**), royal jelly (RJ, sample 15); and (**D**), propolis (PR, sample 16) on HepG2 cell viability by MTT assay. Cells were cultured with different doses of samples for 24 (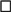), 48 (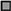), and 72 h (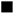). C0, untreated cells. Asterisks indicate significant differences from the control. *** *p* ≤ 0.001, ** *p* ≤ 0.01, and * *p* ≤ 0.05.

Figure 1 shows data on thyme (TH, sample 1) and chestnut (CH, sample 6) honey, royal jelly (RJ, sample 15), and propolis (PR, sample 16) cytotoxicity in HepG2 cells obtained by the MTT method. The studied sample concentrations were 0–250 mg/mL. The results show that as the concentration increases, the percentage of cell survival decreases. Concentrations of thyme and chestnut honey samples (Figure 1A,B) up to 50 mg/mL did not affect the viability of HepG2 cells at 24, 48, and 72 h of treatment (>80%). When the cells were treated with monofloral honey samples at 100 mg/mL for 24 h, no significant differences were observed (*p* > 0.05) compared to the nontreated control cells (>75%). The treatment of cells with this concentration for longer periods of time caused a significant reduction in cell viability (*p* < 0.05). The results obtained in the present study are in agreement with those published by Al Refaey et al. (2021) but for Manuka honey [30]. These authors reported that concentrations up to 50 mg/mL of Manuka honey did not have a significant effect on the viability of HepG2 cells; however, higher concentrations of this type of honey incubated for 48 h had a significant reduction in cell viability [30]. In addition, a significant decrease in cell viability (*p* < 0.05) was observed at 250 mg/mL after treatment with the thyme (42%) and chestnut (30%) samples for 24 h. Recent studies have also evaluated the effect of different honey samples on HepG2 cell viability at similar concentrations and for the same periods of time [31,32]. Halawani (2021) observed a time– and dose–response effect when treating HepG2 cells at 24 h, 48 h, and 72 h with Manuka and Shaoka honeys from Saudi Arabia [32]. Manuka and Shaoka honey samples resulted more cytotoxic at lower concentrations compared to the thyme and chestnut honeys analyzed in the present study [32]. This may be due to the differences in the botanical and geographical origins of the bee product samples that have an influence on their composition and bioactive properties [33]. Royal jelly and propolis (Figure 1C,D) up to a concentration of 100 mg/mL also did not reduce cell viability below 80% when cells were treated for 24 h. However, royal jelly showed a remarkable lower percentage of cell viability (5%) at the concentration of 250 mg/mL at all times tested (24, 48, and 72 h). Propolis was the only sample that at 250 mg/mL did not decrease cell viability (80%) when cells were treated for 24 and 48 h. In contrast, Badria et al. (2018) showed that lower concentrations (0.1 mg/mL) of Bulgarian, Egyptian, and Libyan propolis presented higher cytotoxic activity (7–85%) in HepG2 cells [34]. The authors suggest that the cytotoxic properties of the different propolis samples analyzed in their study can be correlated with their composition (specially flavonoids and total phenolic compounds) that depends on their geographical origin [33,34].

**Figure 2:**
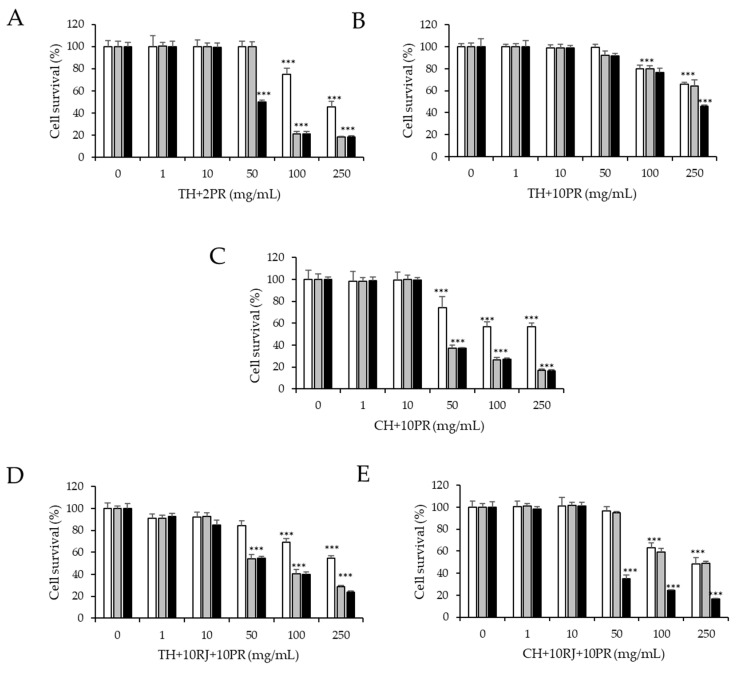
Effect of (**A**), sample 4 (TH + 2PR); (**B**), sample 5 (TH + 10PR); (**C**), sample 10 (CH + 10PR); (**D**), sample 12 (TH + 10RJ + 10PR); and (**E**), sample 14 (CH + 10RJ + 10PR) on HepG2 cell viability by MTT assay. Cells were cultured with different doses of samples for 24 (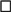), 48 (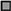), and 72 h (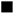). C0, untreated cells. Asterisks indicate significant difference from control. *** *p* ≤ 0.001.

On the other hand, the results of cytotoxicity of the monofloral honey samples enriched with royal jelly or propolis are shown in Figure 2. Concentrations up to 50 mg/mL of TH + 2PR, sample 4 (Figure 2A), did not cause a significant effect (*p* > 0.05) on the cell viability of HepG2 cells. However, concentrations of 100 and 250 mg/mL at 48 and 72 h significantly decreased (*p* < 0.05) the viability to 20%. However, at 24 h, the viability of the cells was above 50%. When propolis was added to the thyme honey at 10% (TH + 10PR, sample 5, Figure 2B), all the concentrations tested at different incubation times did not reduce cell viability below 50%. However, when 10% propolis was added to the chestnut honey (CH + 10PR, sample 10, Figure 2C), concentrations over 50 mg/mL caused a significant decrease (*p* < 0.05) in HepG2 cell viability, reducing it below 40% when cells were treated for 48 and 72 h. When honey samples were enriched with both royal jelly and propolis (Figure 2D,E), concentrations of 100 and 250 mg/mL for 24 h maintained cell viability over 50%, while reduced it to 25–40% and 20–60% when cells were treated during 48 or 72 h with TH + 10RJ + 10PR, sample 12 and CH + 10RJ + 10PR, sample 14, respectively. To the best of our knowledge, there are no previously published studies evaluating the effect of thyme and chestnut honeys enriched with royal jelly and propolis on the viability of HepG2 cells. In line with previous results published for individual monofloral honey samples, the concentrations of honeys enriched with royal jelly and propolis resulted in cytotoxicity to HepG2 cells when incubated at 50 mg/mL for more than 24 h [31]. Nguyen et al. (2021) showed that exposure of HepG2 cells to Manuka and other newly developed honeys (arjuna, guggul, jiaogulan, and olive) for 24 h, 48 h, and 72 h significantly reduced cell viability at 50 and 100 mg/mL [31]. Further research on the effect of monofloral honey enriched with other bee products, such as royal jelly and propolis, on the viability of cellular model is needed to compare the results obtained in the present study.

Due to its wide range of applications in scientific research, the HepG2 cell line has gained popularity. Because this cell line has retained most of the metabolic functions of normal hepatocytes, it is used to study the toxic effects of different substances *in vitro* [35]. In this study, the cell viability assay performed in HepG2 cells was used to select nontoxic concentrations of samples to study their effect on DNA damage. In general, for all tested simples and enriched honey samples and for all incubation times, concentrations up to 10 mg/mL did not result in cytotoxicity to HepG2 cells. However, the tested honey samples, bee products, and their combinations at concentrations over 50 mg/mL affected the viability of HepG2 cells in a time- and dose-dependent manner (Figure 1 and Figure 2). In agreement with our results, the final concentrations of honey samples used to study their effect on DNA damage were below 50 mg/mL, from 0.1 to 10 µg/mL [36].

### 3.2. Analysis of DNA Damage (DNA Strand Breaks) Induced by Thyme and Chestnut Honeys, Royal Jelly, and Propolis in the Alkaline Comet Assay

**Figure 3:**
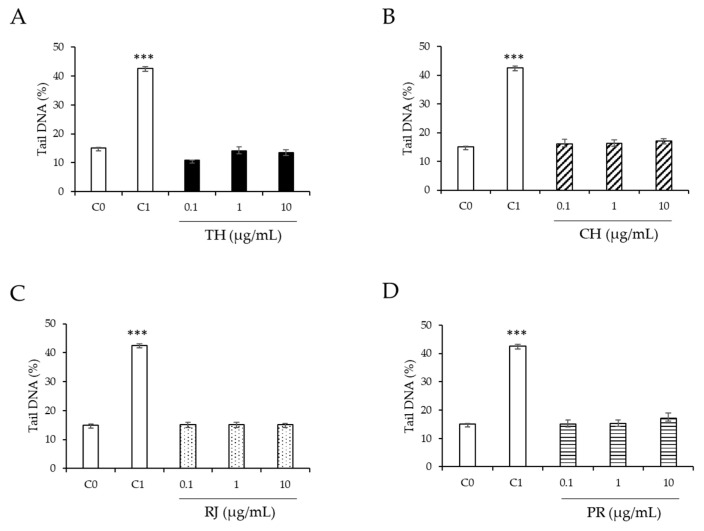
Induction of DNA strand breaks by (**A**), thyme honey (TH, sample 1, 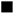); (**B**), chestnut honey (CH, sample 6, 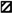); (**C**), royal jelly (RJ, sample 15, 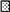); and (**D**), propolis (PR, sample 16, 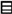) at 0.1–10 µg/mL in human HepG2 cells. C_0_, untreated cells (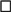). C_1_, cells treated with BaP (100 µM, 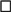). Asterisks indicate significant difference from control (C_0_). *** *p* ≤ 0.001.

None of the honey samples (thyme and chestnut), royal jelly, and propolis at the tested concentrations (0.1–10 µg/mL) caused DNA damage per se (Figure 3) because no significant differences were observed (*p* > 0.05) in the strand breaks when compared to the untreated control cells (C0). Thus, these concentrations were selected for subsequent studies, as these values are more likely to be achieved physiologically. As expected from our previous research [37,38], a significant increase (*p* < 0.05) in strand breaks was observed when BaP 100 μM, used as the positive control (C1), was added to the HepG2 cells (40% tail DNA).

Exposure to environmental toxicants induces oxidative stress, leading to DNA damage induced by ROS [39]. DNA damage can interfere with essential cellular processes, lead to cell damage, and induce mutations that contribute to aging and diseases [40]. According to the European Food Safety Authority (EFSA), genotoxicity studies are used for risk assessments of substances present in food to identify compounds responsible for heritable damage in humans, predict potential genotoxic, and contribute to the understanding of the mechanisms of action of chemical carcinogens [41]. The comet assay is a rapid, sensitive, and relatively simple method recommended for detecting DNA damage at the level of individual cells [42]. In this work, the comet assay was used to evaluate the genotoxic effect of the individual samples (Figure 3). None of them at the tested concentrations produced DNA damage compared to the untreated control cells.

The botanical and the geographical origins of honey contribute to variations in the total polyphenol content and the antioxidant capacity of different kinds of honey [25,43]. To the best of our knowledge, no previous research has reported the effect of monofloral thyme or chestnut honey samples on DNA damage by the comet assay. Other monofloral (rosemary and heather) and heterofloral honey samples were also not genotoxic at concentrations higher than those used in this study [44]. On the other hand, the genotoxic effect of royal jelly and propolis has been previously studied. The genotoxic effect of royal jelly has not been evaluated *in vitro* by the comet assay, but has been studied *in vivo* using animal models. In rats, the administration of Egyptian royal jelly at 100 and 250 mg/kg body weight for 5 days did not induce DNA damage in liver cells [45]. In addition, the peripheral blood cells of mice treated for 24 and 48 h with commercial royal jelly at 150, 300, and 1000 mg/kg did not show significant differences in DNA damage when compared to the control with untreated mice [24].

In agreement with our results, a recent study has shown the nongenotoxic effect of propolis at 10 µg/mL after 24 h treatment of HepG2 cells [22]. In addition, the treatment of other hepatocytes (ZF-L) with higher concentrations of a Brazilian red propolis ethanolic extract (100–500 µg/mL) for 2 h showed no genotoxic effect [46]. In contrast, similar concentrations of propolis to those used in the present study have caused genotoxic effects on fibroblast synovial cells [47]. Gajek et al. (2020) indicated that the compound responsible for this genotoxic effect is caffeic acid phenethyl ester (CAPE), an active component of propolis, when tested on different gastrointestinal neoplastic cell lines [48]. These different effects observed for propolis are expected because the characteristics of this bee product are associated with its region of origin and botanical source that determine its chemical composition [12].

### 3.3. Analysis of DNA Damage (DNA Strand Breaks) Induced by a Simultaneous Treatment of BaP and Thyme and Chestnut Honey Samples and Honey Enriched with Royal Jelly and Propolis in the Alkaline Comet Assay

**Figure 4:**
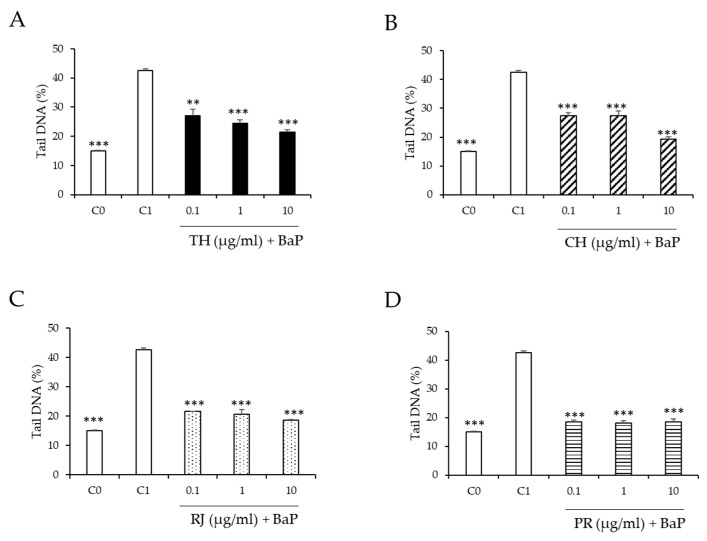
Effect of (**A**), thyme honey (TH, sample 1); (**B**), chestnut honey (CH, sample 6); (**C**), royal jelly (RJ, sample 15); and (**D**), propolis (PR, sample 16) at 0.1–10 µg/mL on BaP-induced DNA strand breaks in HepG2 cells. C_0_, untreated cells (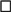). C_1_, cells treated with BaP (100 µM, 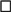). (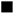) Cells treated with BaP (100 µM) and TH. (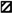) Cells treated with BaP (100 µM) and CH. (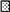) Cells treated with BaP (100 µM) and RJ. (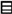) Cells treated with BaP (100 µM) and PR. Asterisks indicate significant difference from control (C_1_). *** *p* ≤ 0.001 and ** *p* ≤ 0.01.

The effect of thyme and chestnut honeys, royal jelly, and propolis on BaP-induced DNA damage in HepG2 cells is shown in Figure 4. All samples tested at all concentrations used (0.1–10 µg/mL) decreased the genotoxic effect of BaP 100 µM. Among the studied samples, propolis showed the highest percentage of reduction (56.3–57.7%) at all tested concentrations (Figure 4D). Our previous research indicated that propolis possesses the highest antioxidant properties compared to the other studied bee products [25], which may be the reason why propolis has shown the greatest potential for reducing induced DNA damage. In addition, royal jelly and chestnut and thyme honeys at 10 µg/mL significantly reduced DNA strand breaks (*p* < 0.05) induced by BaP in a 56.3%, 55%, and 49.7%, respectively.

**Figure 5:**
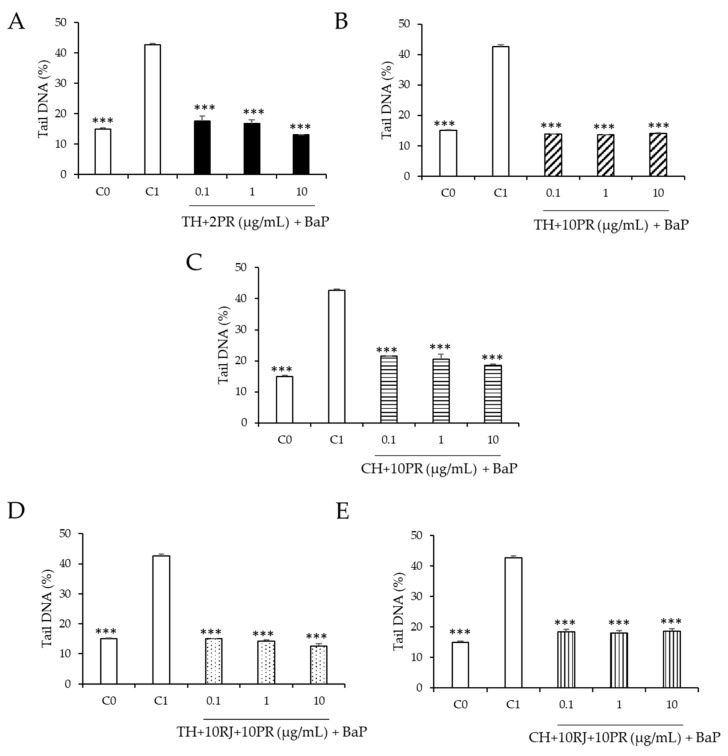
Effect of (**A**), sample 4 (TH + 2PR); (**B**), sample 5 (TH + 10PR); (**C**), sample 10 (CH + 10PR); (**D**), sample 12 (TH + 10RJ + 10PR); and (**E**), sample 14 (CH + 10RJ + 10PR) at 0.1–10 µg/mL on BaP-induced DNA strand breaks in HepG2 cells. C_0_, untreated cells (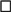). C_1_, cells treated with BaP (100 µM, 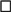). (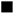) Cells treated with BaP (100 µM) and TH + 2PR. (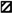) Cells treated with BaP (100 µM) and TH + 10PR. (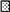) Cells treated with BaP (100 µM) and TH + 10RJ + 10PR. (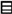) Cells treated with BaP (100 µM) and CH + 10PR. (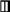) Cells treated with BaP (100 µM) and CH + 10RJ + 10PR. Asterisks indicate significant difference from control (C_1_). *** *p* ≤ 0.001.

**Figure 6:**
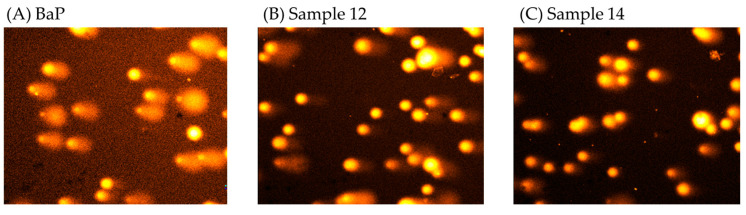
Representative images (10×) of the effect of (**A**), BaP (100 µM); (**B**), sample 12 (TH + 10RJ + 10PR); and (**C**), sample 14 (CH + 10RJ + 10PR) at 1 µg/mL on BaP-induced DNA strand breaks in HepG2 cells.

The protective effect of honey samples enriched with royal jelly or propolis on BaP-induced DNA damage is shown in Figure 5. All mixtures of thyme or chestnut honey samples with royal jelly or propolis showed a greater protective effect against BaP than the samples alone. The thyme and chestnut honey samples enriched with 10% royal jelly and 10% propolis (Figure 5D,E) showed the highest protective effect against BaP (70.4% and 69.4%, respectively). Figure 6 shows the typical comet assay images after treatment with BaP (100 µM) and the absence of comets after exposure to BaP and samples 12 and 14. Thyme honey enriched with 2 or 10% propolis (Figure 5A,B) had very similar values (69% and 67.8%) as did the chestnut honey sample enriched with 10% propolis (67.8%). This is the first time the chemoprotective effect of enriched honey mixtures has been evaluated on induced DNA damage. In this study, the observed reduction in induced DNA damage by BaP by both honey samples alone and mixed with other bee products was about two times higher than that reported for other monofloral (rosemary and heather) and heterofloral kinds of honey [44]. Different chemoprotective effects have been observed depending on the botanical origin of honey samples. Rosemary honey at 0.1–10 mg/mL decreased DNA strand breaks induced by BaP in a dose-dependent manner (19–25%), and heather honey prevented DNA strand breaks only at the lowest concentration (0.1 mg/mL, 15%) used in that study [44]. Heterofloral honey reached a maximum decrease in DNA strand breaks at the highest concentration used of 10 mg/mL (34%). Because an artificial honey sample did not show any protective effects against DNA damage induced by BaP, polyphenols might be, at least partially, the potential chemopreventive agents present in honey that protect against damage induced by BaP [44]. In fact, it has been described that due to their polyphenolic profile, monofloral honey samples have a significant antioxidant capacity, as well as antidiabetic, antimicrobial, and anticancer properties [49].

Even though honey is recognized as one of the first known functional foods and has been widely used in traditional medicine for centuries (apitherapy) [7,50], the scientific community is still interested in exploring further health-promoting properties honey may possess [50]. In order to clarify to honey consumers what honey’s total potential is, a newly joined property named “Power of Honey” considering honey’s antibacterial and antioxidant properties have been recently defined [50]. This novel approach successfully predicted the greater Power of Honey in forest honey compared to acacia, sunflower, and meadow honey samples [50]. The high antioxidant capacity of honey and the other bee products studied in the present work plays an important physiological role due to its capacity to scavenge hydroxyl and superoxide radicals related to cell and DNA damage and related diseases [12]. In this context, genotoxicity tests are also useful for assisting in the discovery of genoprotective substances for DNA mutation prevention [46]. In the present study, all tested individual samples at all concentrations used (0.1–10 µg/mL) decreased the genotoxic effect produced by BaP (Figure 4). In addition, the mixtures of thyme honey or chestnut honey with royal jelly or propolis showed a greater protective effect against BaP than the samples alone (Figure 6).

The observed protective effects may be associated with the phenolic content and antioxidant capacity of the studied samples that have been recently reported in our previous study [25]. The antioxidant potential of these honey samples has been confirmed by the analysis of their total phenolic content analyzed using the Folin–Ciocalteu reagent and antioxidant capacity by three different assays (DPPH, ABTS, and ORAC) to consider the complexity of the food matrix, the chemical diversity of antioxidants, and the different mechanisms of oxidative processes. A significant positive correlation was observed between total phenolic compounds and the antioxidant capacity analyzed by the three methods, suggesting that the antioxidant capacity could be attributed, at least partially, to the phenolic compounds present in the samples [25]. Our previous research indicated that mixtures of honey with 10% of propolis increased the value of total phenolic content and antioxidant capacity compared to natural honey and therefore were chosen to be analyzed in the present study. In addition, these samples have shown the potential to reduce ROS production in the same human hepatoma cells (HepG2 cell line) [25]. Other Spanish honey samples, such as heather, rosemary, and heterofloral, have also shown the ability to significantly decrease intracellular ROS levels in a time-dependent manner in HL-60 cells [51]. Chestnut honey enriched with 10% RJ and 10% PR (sample 14) was the most effective in the reduction in intracellular ROS, and it even improved the results obtained for N-acetyl-L-cysteine used as an antioxidant control [25]. In this study, sample 14, together with sample 12 (TH + 10RJ + 10PR), was also the most effective in preventing induced DNA damage (Figure 6).

Honey is a good source of natural antioxidants whose activity is mainly due to the presence of phenolic compounds, although amino acids, proteins, vitamins, and carotenoid derivatives also contribute to the antioxidant capacity of this food product [25,43]. Polyphenols, and more specifically flavonols and flavanols, such as quercetin and myricetin, and catechin and epicatechin, respectively, protect human-derived cells against DNA strand breaks and oxidative DNA damage effects induced by BaP [38]. These compounds have been found both in thyme [52,53,54] and chestnut [49,55] honey samples, and therefore, the observed protective effect against induced DNA damage can be associated, at least partially, to these compounds. Polyphenols are the main constituents of propolis that have health-promoting properties, and propolis has been reported as the bee product containing the highest number of phenolic compounds [56]. Polyphenols, such as quercetin [57] and catechin [12], that have shown protective effects against this specific carcinogen, have been reported in this bee product. In addition, CAPE, known as a key anticancer component [58], has been ascribed as the most valuable biologically active compound in propolis for its several biological and pharmacological properties [56]. This compound has shown to effectively prevent cellular DNA damage induced by overloaded iron through decreasing the labile iron pool levels in HeLa cells [59]. Polyphenols are also present in royal jelly [25,60], but proteins are the most abundant components (50% dry weight), with major royal jelly proteins (MRJPs) being the most important (80–90%) [14]. These proteins also protect against DNA damage induced by ROS, acting as scavengers against superoxide anions and hydroxyl radicals [61].

According to Patra et al. (2021), dietary phytochemicals are “non-nutritive, relatively non-toxic, disease preventive phytoconstituents that are safe to consume”, and including them in a healthy diet can prevent about 30% of carcinogenesis [62]. Dietary polyphenols have an important role in cancer prevention by their antiproliferative and apoptotic properties, among others [62]. In this context, three types of crude Spanish commercial honey samples (heather, rosemary, and heterofloral) induced apoptosis in a concentration- and time dependent-manner. Heather and heterofloral honey samples possessed the highest phenolic content and were the most effective to induce apoptosis in human peripheral blood promyelocytic leukemia cells (HL-60 cells) [51]. These same honey samples have shown protective effects against acrylamide-induced cytotoxicity [63]. Because an artificial honey sample did not mitigate the cytotoxic effect induced by acrylamide, the protective effect of these honeys may be attributed to their polyphenolic content and not to the sugar constituents present in this food matrix [63]. In addition to protecting against induced DNA damage, the chestnut honey studied in the present work may also present anticancer effects by promoting apoptosis due to the presence of 3–2′-pyrrilonidinyl-kynurenic acid (3-PKA-L), an alkaloid usually present in this monofloral honey, that has shown proapoptotic properties [64]. Further studies demonstrating the potential of monofloral honey samples as healthy dietary ingredients with chemopreventive properties are needed to support local honey production and respond to the increasing honey demand of consumers derived from the COVID-19 pandemic.

## 4. Conclusions

In this work, we proved the noncytotoxic and nongenotoxic characteristics of monofloral honey samples enriched with royal jelly and propolis in order to give more information related to the safety and chemoprotective potential of these enriched products. Our results indicate, for the first time, that thyme and chestnut honeys, royal jelly, propolis, and their combination protect human cells from DNA strand breaks induced by BaP. Thyme and chestnut honey samples enriched with 10% royal jelly and 10% propolis showed the highest protective effect against food mutagen-induced DNA damage in HepG2 cells. Polyphenols present in these samples and their antioxidant capacity are partially responsible for the observed chemoprotective effect.

## Figures and Tables

**Table 1:** Honey and bee product samples.

Samples	Scientific and Common Names	Family	Geographic Region	Type
Chestnut honey	*Castanea sativa* Chestnut	Fagaceae	Spain (Toledo)	Monofloral
Thyme honey	*Thymus* spp. Thyme	Lamiaceae	Spain (Zamora)	Monofloral
Royal jelly	-	-	France	-
Propolis tincture *	-	-	Spain (Zamora)	-

* Propolis extract dissolved in 70% organic ethanol.

**Table 2:** Honey and bee product samples and mixtures selected from a previous work [25].

Code	Description	Sample nº
TH	Thyme honey	1
TH + 2PR	Thyme honey + 2% propolis	4
TH + 10PR	Thyme honey + 10% propolis	5
CH	Chestnut honey	6
CH + 10PR	Chestnut honey + 10% propolis	10
TH + 10RJ + 10PR	Thyme honey + 10% royal jelly + 10% propolis	12
CH + 10RJ + 10PR	Chestnut honey + 10% royal jelly + 10% propolis	14
RJ	Royal jelly	15
PR	Propolis	16

## Data Availability

All relevant data are within the manuscript, and individual data can be accessed from the corresponding author upon reasonable request.
